# Elevated platelet–leukocyte complexes are associated with, but dispensable for myocardial ischemia–reperfusion injury

**DOI:** 10.1007/s00395-022-00970-3

**Published:** 2022-11-16

**Authors:** Christopher Starz, Carmen Härdtner, Maximilian Mauler, Bianca Dufner, Natalie Hoppe, Katja Krebs, Carolin Anna Ehlert, Julian Merz, Timo Heidt, Peter Stachon, Dennis Wolf, Christoph Bode, Constantin von zur Muehlen, Wolfgang Rottbauer, Meinrad Gawaz, Daniel Duerschmied, Florian Leuschner, Oliver Borst, Dirk Westermann, Ingo Hilgendorf

**Affiliations:** 1 Department of Cardiology and Angiology, University Heart Center Freiburg-Bad Krozingen, Faculty of Medicine, Faculty of Medicine, University of Freiburg, Freiburg, Germany; 2 Department of Internal Medicine II, University of Ulm, Ulm, Germany; 3 Department of Cardiology and Cardiovascular Medicine, University Hospital Tuebingen, Tuebingen, Germany; 4 Department of Cardiology and Angiology, Faculty of Medicine, University of Heidelberg, Mannheim, Germany; 5 Department of Medicine III, Faculty of Medicine, University of Heidelberg, Heidelberg, Germany; 6 InflaMyoCard Research Consortium, The Baden-Wuerttemberg Ministry of Science, Research and Arts, Stuttgart, Germany; 7 Institute for Experimental Cardiovascular Medicine, Faculty of Medicine, University Heart Center Freiburg-Bad Krozingen, University of Freiburg, Freiburg, Germany

**Keywords:** Platelet–leukocyte complexes, Myocardial infarction, Ischemia/reperfusion injury, Inflammation, P-selectin

## Abstract

**Aims:**

P-selectin is an activatable adhesion molecule on platelets promoting platelet aggregation, and platelet–leukocyte complex (PLC) formation. Increased numbers of PLC are circulating in the blood of patients shortly after acute myocardial infarction and predict adverse outcomes. These correlations led to speculations about whether PLC may represent novel therapeutic targets. We therefore set out to elucidate the pathomechanistic relevance of PLC in myocardial ischemia and reperfusion injury.

**Methods and results:**

By generating P-selectin deficient bone marrow chimeric mice, the post-myocardial infarction surge in PLC numbers in blood was prevented. Yet, intravital microscopy, flow cytometry and immunohistochemical staining, echocardiography, and gene expression profiling showed unequivocally that leukocyte adhesion to the vessel wall, leukocyte infiltration, and myocardial damage post-infarction were not altered in response to the lack in PLC.

**Conclusion:**

We conclude that myocardial infarction associated sterile inflammation triggers PLC formation, reminiscent of conserved immunothrombotic responses, but without PLC influencing myocardial ischemia and reperfusion injury in return. Our experimental data do not support a therapeutic concept of selectively targeting PLC formation in myocardial infarction.

**Supplementary Information:**

The online version contains supplementary material available at 10.1007/s00395-022-00970-3.

## Introduction

Improving outcomes and preserving cardiac tissue in patients with acute myocardial infarction (AMI) represents one of the most challenging unmet medical needs in cardiovascular care to date.

The gold standard in treating AMI is the timely restoration of myocardial blood flow to minimize ischemic injury, but it comes at a price: Reperfusion injury. Myocardial ischemia and reperfusion (I/R) injury manifests itself in reperfusion arrhythmia, myocardial stunning, microvascular obstructions, and cardiomyocyte cell death, responsible for up to 50% of the final infarct size [59]. Unfortunately, most therapeutic interventions directed against myocardial I/R injury have failed to show benefits in clinical trials to date [9, 46]. A rare exception may be P-selectin antagonism in patients undergoing percutaneous coronary intervention for non-ST-segment elevation MI, which blunted post-procedural troponin and creatine kinase-myocardial band rises in blood as surrogates of reduced myocardial damage [53], but clinical endpoints have not been tested in a large follow-up trial.

The formation of platelet–leukocyte complexes (PLC) largely depends on the binding of platelet-derived P-selectin to leukocyte-derived P-selectin glycoprotein ligand 1 (PSGL-1) [14, 27, 42, 46]. In platelets, P-selectin is stored intracellularly in alpha granules and is transferred to the cell surface upon platelet activation [39]. Increased frequencies of circulating PLC, platelet–monocyte (PMC) and platelet–neutrophil complexes (PNC) in particular, have been recorded in patients with AMI or unstable angina [18, 38, 43]. Elevated numbers of circulating PMC represent early markers of AMI [38]. Their frequencies correlate with troponin levels [60] and predict future cardiovascular events within 2 years following AMI [61]. Experimental studies, ex vivo*,* suggest that platelet binding to monocytes and neutrophils can stimulate proinflammatory responses [11, 14] and facilitate microvascular obstructions in vivo [22, 24, 40]. Taken together, PLC are postulated to contribute directly to myocardial I/R injury, and to represent a therapeutic target [25, 32]. In this study, we challenge the hypothesis of a direct causal involvement of PLC in myocardial I/R injury in vivo*.*

## Methods

### Animals

Male C57BL/6J (WT) and P-selectin-deficient B6;129S7-Selp^tm1Bay/J^ (KO) mice were provided by the Jackson Laboratories (Maine, USA). Eight-week-old mice from both genotypes were lethally irradiated and reconstituted with 10^7^ WT or KO bone marrow cells, respectively. Hereby, we created three different types of bone marrow chimeras: WT in WT (WTWT), KO in WT (KOWT), and WT in KO (WTKO). Euthanasia was performed by CO_2_ inhalation. All animal procedures were approved by the Animal Care Committee of the University Freiburg and regional councils.

### Blood collection and cell counts

Murine blood was obtained by facial vein puncture and collected in 10 µL Enoxaparin per 100 µL blood. Larger volumes were gained after euthanasia with CO_2_ and cardiac cannulation. Leukocytes were counted manually, using a Neubauer counting chamber and diluting with red blood cell lysis buffer 1:20 (Thermo Fisher Scientific, Waltham, MA, USA). Standardized blood counts for leukocytes, platelets, and erythrocytes were performed by the University Central Laboratory.

### Plasma

For plasma generation, blood was centrifuged in two steps at 1500×*g* at room temperature for 5 min, collecting the supernatant. Plasma was immediately shock frozen in liquid nitrogen and stored at − 20 °C until further processing.

### Platelet-rich plasma (PRP)

Murine blood was diluted 1:1 with Tyrode’s buffer and 0.3% enoxaparin, centrifuged, and the supernatant transferred the supernatant (PRP) to a new reaction tube. PRP was always kept at 37 °C. For measuring surface marker expression, PRP was stimulated with 10 µL 200 µM adenosine diphosphate (ADP) (Merck KGaA, Darmstadt, Germany) for 30 min and analyzed by flow cytometry.

### Flow cytometry

Heparinized blood was diluted 1:6 with PBS. Diluted blood was used for antibody staining. After 15 min of incubation at 37 °C, blood was lysed and fixed with BD^®^ Lysis/Fix™ (BD Bioscience, Becton Dickinson, Heidelberg, Germany).

Heart tissues were enzymatically digested [collagenases I and IX, hyaluronidase as well as DNAse I (Sigma-Aldrich, St. Louis, Missouri, USA)] and heart cell suspensions are processed through a 40 µm strainer using PBS/ 0.5% BSA/1% FCS. Cells were stained with specific antibodies. For endothelial cell isolation, we minced aortas and digested them with collagenase IV (Worthington Biochemical Corporation, Lakewood, NJ, USA) and DNAse I (Sigma-Aldrich, St. Louis, Missouri, USA). Antibodies used are listed in Supplemental Table 1. For ROS detection, blood cells were incubated with 2′,7′-dichlorofluorescin diacetate (DCFDA, Merck KGaA, Darmstadt, Germany) prior to antibody staining.

Flow cytometry analysis was performed with a BD FACS Canto II (BD Bioscience, San Diego, CA, USA).

### Imaging flow cytometry

Imaging flow cytometry analysis of murine blood samples was performed at the *Image Stream Core Facility* (Tübingen, Germany) with an *Imagestream® X Mark II* Imaging Flow Cytometer (Merck KGaA, Darmstadt, Germany). In this approach, fluorescent antibody surface staining is combined with intracellular phalloidin staining of actin following cell permeabilization with 0.1% Triton X (both obtained from Merck KGaA, Darmstadt, Germany).

Antibodies used are listed in the supplemental methods (Supplemental Table 1).

### Histology

The infarcted areas of isolated hearts were embedded in TissueTek^®^ O.C.T.™ (OCT) (Sakura Fintek, Tokyo, Japan), frozen at − 20 °C and cut into 4 µm sections. Neutrophils were identified by anti-Ly6G rat anti-mouse antibody binding (BD, New Jersey, USA). For overall tissue, morphology hematoxylin and eosin staining was performed.

### High-sensitive troponin T (hsTnT) levels

Plasma was diluted 1:15 with PBS, and hsTnT levels were determined by automated electrochemiluminescence analysis according to the detailed protocol from Andrassy et al. [1].

### Myocardial ischemia–reperfusion (I/R) injury

In anesthetized mice, the left anterior descending (LAD) coronary vessel was obstructed for 35 min and then reperfused again. Sham operated mice underwent only thoracotomy followed by 35 min of anesthesia [buprenorphine (0.1 mg/kg) and isoflurane inhalation]. 24 h after I/R, mice were euthanized, hearts were flushed with isotonic saline and excised for further processing. Postoperative analgesia was performed with buprenorphine (0.1 mg/kg) subcutaneously every 6–8 h.

### Echocardiography

Transthoracic echocardiography was performed on isoflurane anesthetized mice with a Vevo 3100 ultrasound device with an MX550D linear probe (Visualsonics, Toronto, Canada). Left-ventricular ejection fraction was measured using the Simpson method.

### Peritonitis model

For sterile peritonitis, mice were injected with 2 mL 4% thioglycollate intraperitoneally (i.p.) (Merck KGaA, Darmstadt, Germany). After 4 h, mice were euthanized, and peritoneal lavage was performed by injecting and aspirating 5 mL PBS. Cells were washed, counted (Neubauer counting chamber), and stained for flow cytometry analysis.

### Intravital microscopy

Mice were stimulated by injecting 10 g/kg tumor necrosis factor alpha (TNFα) i.p. 4 h prior to intravital microscopy. Following ketamine (100 mg/kg) and xylazine (100 mg/kg) anesthesia and rhodamine injection (i.p.), we exposed the mesenteric venules and recorded up to 12 30 s clips per mouse.

Rhodamine intravital microscopy was performed with a Axiotech vario 100 HD microscope, a AxioCam Mrm digital camera, and Colibri 2 light source (all Zeiss, Oberkochen, Germany).

For two-color intravital microscopy, mice were injected intravenously with X488/anti-GPIbβ (emfret Analytics, Eibelstadt, Germany) to label platelets and with anti-Gr1-PE (Bio Legend) to label neutrophils and classical monocytes 15 min prior to image recording. Analysis was performed with a Nikon^®^ FN-S2N fluorescence microscope, Hamamatsu Orca-05G digital camera and Nikon Intenslight C-HGFIE.

### TTC

After excision of the heart and proximal aorta, the LAD was occluded again at the site of the suture. The hearts were perfused with Monolight blue 5% followed by further rinsing with 0.9% NaCl and rapid freezing. Frozen hearts were sliced and incubated for 5 min at 37 °C in 2,3,5-triphenyl-tetrazolium chloride (TTC). After 24 h at 4 °C, heart slices were photo-documented and analyzed for area at risk (AAR) and infarct area.

### qRT-PCR

Frozen infarcted heart tissue was defrosted, homogenized, and processed according to the instructions of the Qiagen RNeasy^®^ Mini Kit (Qiagen, Düsseldorf, Germany). Hereby generated RNA was further transcribed into cDNA using the High-Capacity cDNA Reverse Transcription Kit (ThermoFisher Scientific Inc., Waltham, USA) according to the manufacturer's instructions. cDNA transcription was performed with a Oviation PicoSL WTA V2 System (NuGEN, San Carlos, CA, USA). qRT-PCR was performed using Taq-Man probes and a CFX96 Touch Real-Time PCR System (Bio-Rad Laboratories, Hercules, CA, USA). Taq-Man probes used are listed in supplemental Table 2. Target gene expression levels were calculated using the 2-ΔΔ*CT* method with β-actin as the house keeping gene. Results per mouse and target gene were interpreted and visualized relative to mean target gene expression levels in WTWT chimeras.

### Statistical analysis

Results are shown as Mean ± standard error of the mean (SEM). Results are declared as significant with a *p* value ≤ 0.05. The choice of statistical calculation method was based on criteria such as normal distribution and the number of groups to be compared. When comparing two normally distributed groups with mutually independent measurements, the unpaired t test (also Student’s *t* test) was applied. Multiple *t* test was used to compare the PCR data. Due to the multiple testing, a Bonferroni correction was applied. If there were more than two groups to be compared with measurements that were independent of each other, statistical significance was tested with the one-way analysis of variance (ANOVA) with Holm–Sidak posthoc testing. The Kruskal–Wallis test was used as a non-parametric test with Dunns post hoc testing. Statistical analysis was performed using Excel 2010 (Microsoft, Redmond, Washington, USA) and Prism 6 (GraphPad Software, San Diego, California, USA). When considering more than one influencing variable, statistical significance was tested using the two-way analysis of variance (ANOVA), assuming a normal distribution. Sidak's post hoc test was also applied. Bivariate correlations of PMC or PNC levels with plasma troponin levels were calculated using GraphPad Software 2021 (San Diego, CA, USA).

## Results

### PMC and PNC surge in blood following myocardial ischemia and reperfusion in mice

**Fig. 1:**
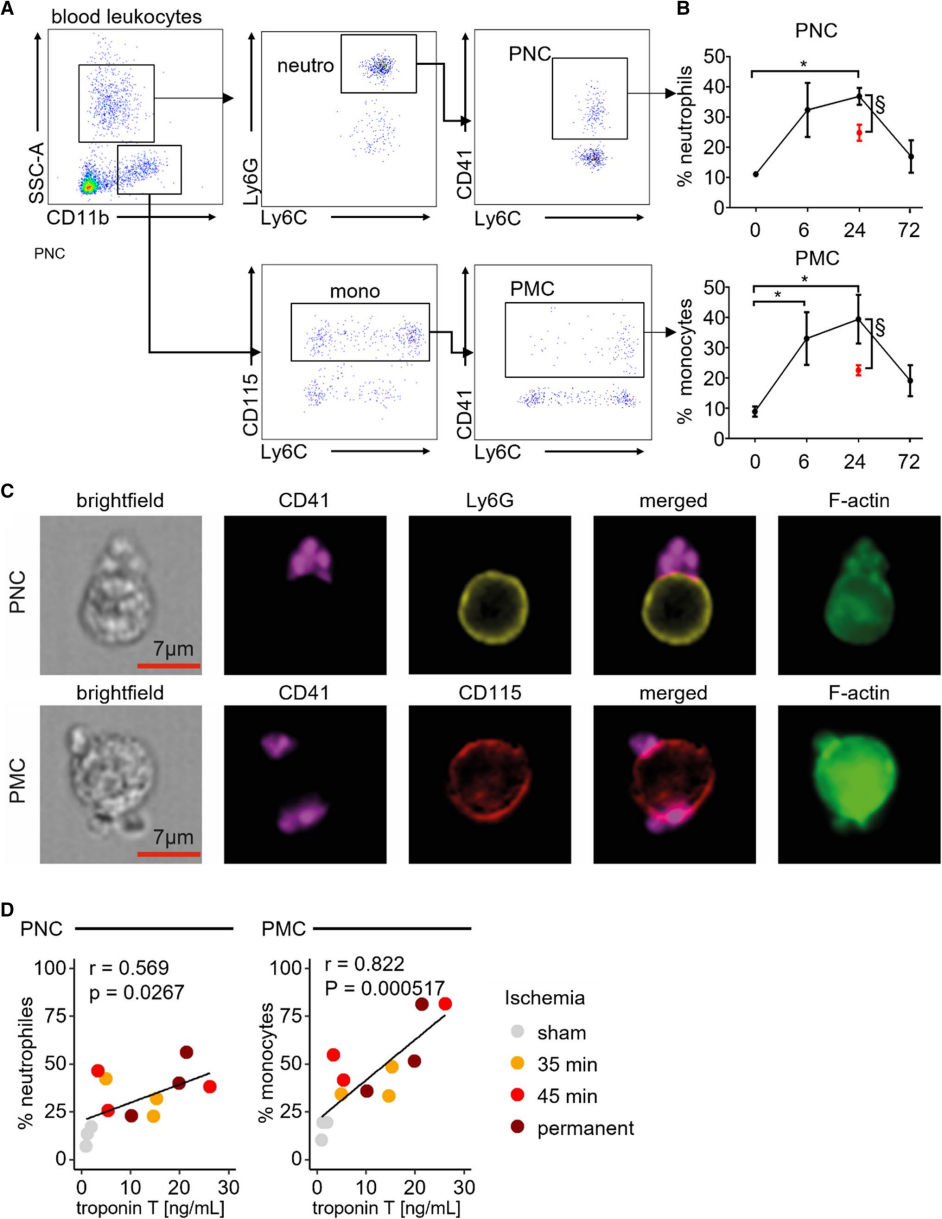
Platelet–leukocyte complexes surge in peripheral blood post-myocardial ischemia and reperfusion injury (I/R). **A** Representative dot plots depicting blood neutrophils, monocytes, platelet–neutrophil complexes (PNC), and platelet–monocyte complexes (PMC), respectively. **B** Time course for PNC (top) and PMC (bottom) fractions in blood before (*t* = 0) and after myocardial ischemia (35 min) with reperfusion over 72 h (black data points). *p < 0.05 denote statistically significant changes before vs after myocardial I/R (*) *n* = 4 per time point, Kruskal–Wallis test, Dunn’s multiple comparison, ^§^*p* < 0.05 denote statistically significant changes between myocardial I/R (black) and sham (red) at 24 h of reperfusion, *n* = 4 per group, Mann–Whitney. **C** Representative ImageStreamX image PNC and PMC defining platelets as CD41 + , neutrophils as Ly6G + , monocytes as CD115 + and F-actin enrichment at the contact zone. **D** Correlation of plasma troponin and blood PMC or PNC levels following 35 min and 45 min of myocardial ischemia with reperfusion or permanent LAD ligation 24 h post-surgery. *p < 0.05 denote statistically significant changes, determination of correlation by Pearson’s *r*

We identified and quantified complexes of platelets with monocytes and neutrophils in blood using fluorescent antibody staining and flow cytometry in mice (Fig. 1A). PMC and PNC formation was visualized and confirmed by imaging flow cytometry (Fig. 1C). Frequencies of PMC and PNC circulating in blood following 35 min of myocardial ischemia with reperfusion for 6, 24, and 72 h were compared to those quantified in healthy and sham operated mice (Fig. 1B). PMC and PNC formation peaked and more than tripled after 24 h of reperfusion and returned to pre-procedural levels at the 72 h reperfusion time point (Fig. 1B). PNC and PMC fractions correlated positively with troponin T levels measured 24 h post 35 min or 45 min of myocardial ischemia with reperfusion, or post-permanent LAD ligation, respectively (Fig. 1D). These data are in line with reports in patients with AMI, in which PLC frequencies increase transiently after coronary revascularization and in which troponin T levels correlate with PMC frequencies [3, 38]. Since 35 min of myocardial I/R injury induced reproducible and sufficient troponin elevations with significant increases in circulating PLC numbers, we used this model to explore the functional role of PLC in myocardial I/R injury.

### Platelets and leukocytes within PLC are relatively activated compared to freely circulating cells

First, we set out to quantify relative cell surface protein expression levels of leukocyte and platelet activation markers in platelet-bound monocytes and neutrophils compared to unbound blood cells in C57Bl6 mice (Suppl. Fig. 1A). Direct comparison was complicated by the fact that CD41 expression levels on platelets within PLC appeared on average lower than levels detected in non-leukocyte-bound platelets. Since CD41 is constitutively expressed on platelets, as confirmed by ex vivo ADP stimulation of whole blood (Suppl. Fig. 1B) and in line with image flow cytometry data (Fig. 1C), we concluded that only one or few platelets bind per leukocyte, while higher numbers of non-leukocyte-bound platelets can form aggregates among each other cumulatively expressing higher mean levels of CD41 (Suppl. Fig. 1A, C). To control for the bias introduced by differences in platelet numbers forming platelet–leukocyte and platelet–platelet aggregates, we normalized platelet activation marker expression levels in PLC and non-leukocyte-bound platelets to the mean fluorescence intensity (MFI) levels of CD41 in the respective populations. Higher levels of P-selectin (CD62P), glycoproteins (GP) Ib, IIb/IIIa and VI were detected on platelets forming complexes with monocytes and neutrophils compared to those not bound to leukocytes. Reactive oxygen species were highest in leukocytes binding platelets compared to non-complexed leukocytes and platelets (Suppl. Fig. 1D). These data indicate that activated platelets exposing high levels of surface P-selectin preferentially engage in PLC formation.

### Inhibiting PLC formation by deleting P-selectin on platelets

**Fig. 2:**
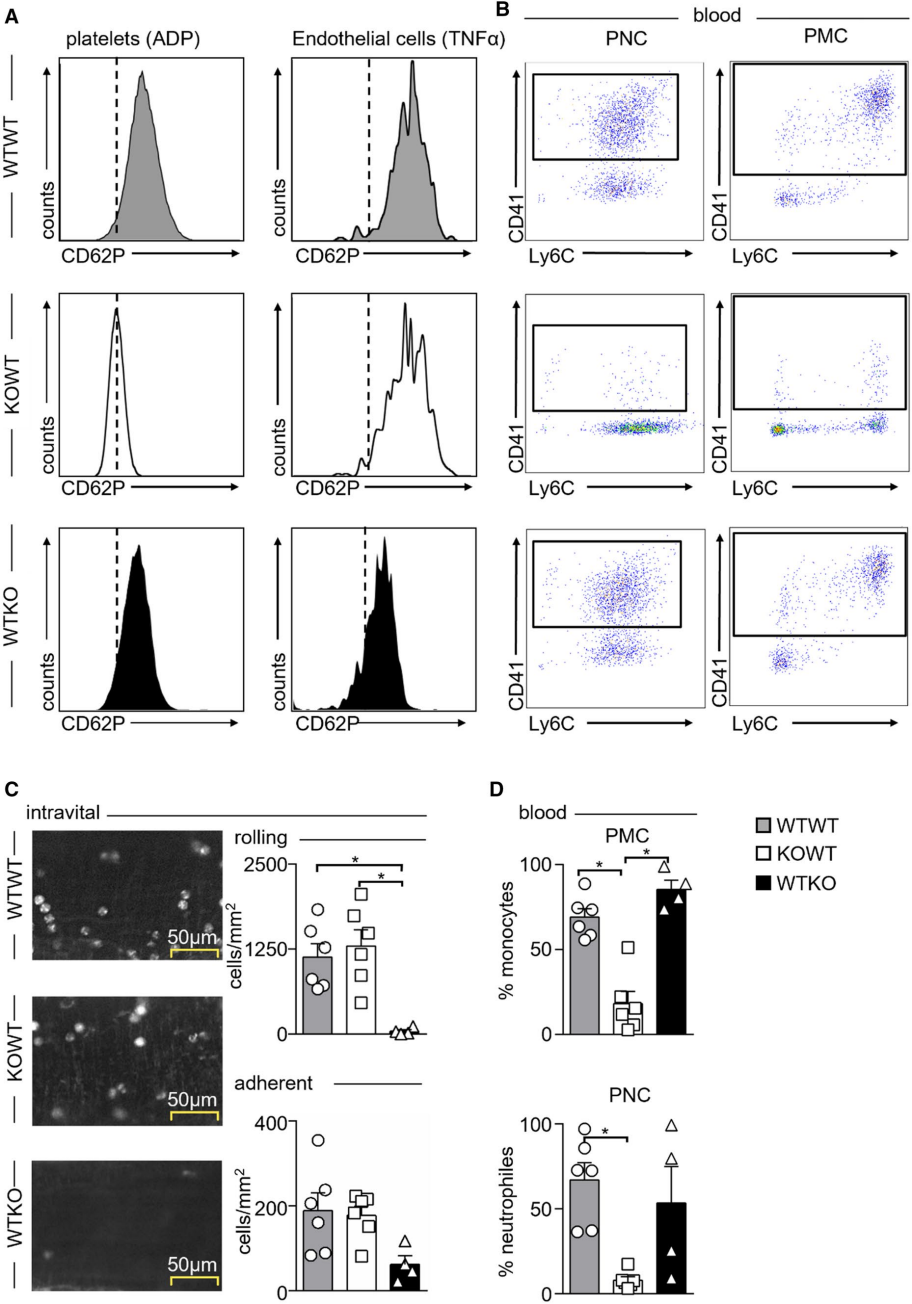
Surface expression of P-selectin (CD62P) and formation of platelet–leukocyte complexes in irradiated bone marrow chimeras, and its influence on leukocyte rolling and adhesion. Wild-type (WT) and P-selectin-deficient (KO) mice were lethally irradiated and reconstituted with either WT bone marrow in WT recipients (WTWT), KO bone marrow in WT recipients (KOWT), or WT bone marrow in KO recipients (WTKO). **A** Representative histograms showing selective loss of CD62P expression on platelets in KOWT chimeras and loss of CD62P expression on endothelial cells in WTKO chimeras. **B** Representative dot plots showing that selective loss of CD62P on platelets in KOWT chimeras reduces both platelet–neutrophil (PNC) and platelet–monocyte complexes (PMC) in blood. **C** Intravital microscopy of mesenteric venules in WTWT, KOWT, and WTKO chimeras 4 h post-intraperitoneal (i.p.) TNFα (200 ng) injection. Representative images of rhodamine-labeled leukocytes within the venules are shown on the left. Quantification of rolling and adherent leukocyte numbers are shown on the right. Results are presented as mean ± SEM, *n* = 6 for WTWT, KOWT and *n* = 4 for WTKO. *p < 0.05 denote statistically significant changes between groups as indicated using Kruskal–Wallis with Dunn’s multiple comparison test. **D** Quantification of blood PMC and PNC in WTWT, KOWT, and WTKO chimeras post-TNFα i.p. stimulation. Results are presented as mean ± SEM, *n* = 6 WTWT and KOWT, *n* = 4 WTKO. *p < 0.05 denote statistically significant changes between groups using Kruskal–Wallis with Dunn’s multiple comparison test

To test the functional impact of PLC in a mouse model of myocardial I/R, we had to generate a PLC-deficient model first. P-selectin (CD62P) is exclusively expressed on activated platelets and endothelial cells (EC) [7] and binds to PSGL-1 on leukocytes mediating PLC formation and endothelial rolling and tethering [16, 34]. Therefore, a global P-selectin-deficient mouse model is not suitable to test the role of PLC selectively, prompting us to explore the potential of irradiation bone marrow chimeras. We generated three types of chimeras: (I) Irradiated C57Bl6 wild-type (WT) mice reconstituted with WT bone marrow cells (WTWT group), (II) irradiated WT mice reconstituted with P-selectin deficient (KO) bone marrow cells (KOWT group), and (III) irradiated KO mice reconstituted with WT bone marrow cells (WTKO group). In the WTWT irradiation control group, P-selectin is readily detectable on ADP-stimulated platelets and TNFα-stimulated aortic endothelial cells, whereas in KOWT mice, P-selectin is lost on platelets but not endothelial cells, and vice versa in WTKO mice (Fig. 2A). PLC formation was evaluated by blood flow cytometry, and leukocyte rolling and adhesion to the vessel wall were imaged by intravital microscopy of mesenteric venules in the three groups (Fig. 2B–D). Both PNC and PMC fractions were reduced by at least 70% in KOWT mice in which P-selectin is lost selectively in platelets, but not in WTKO chimeras. In contrast, leukocyte rolling along the vessel wall was impaired in WTKO chimeras in which P-selectin is lost on endothelial cells. Intriguingly, leukocyte rolling and attachment remained unaffected in KOWT chimeras despite the lack of PLC.

### Lack of PLC does not impair leukocyte infiltration to sites of sterile inflammation

**Fig. 3:**
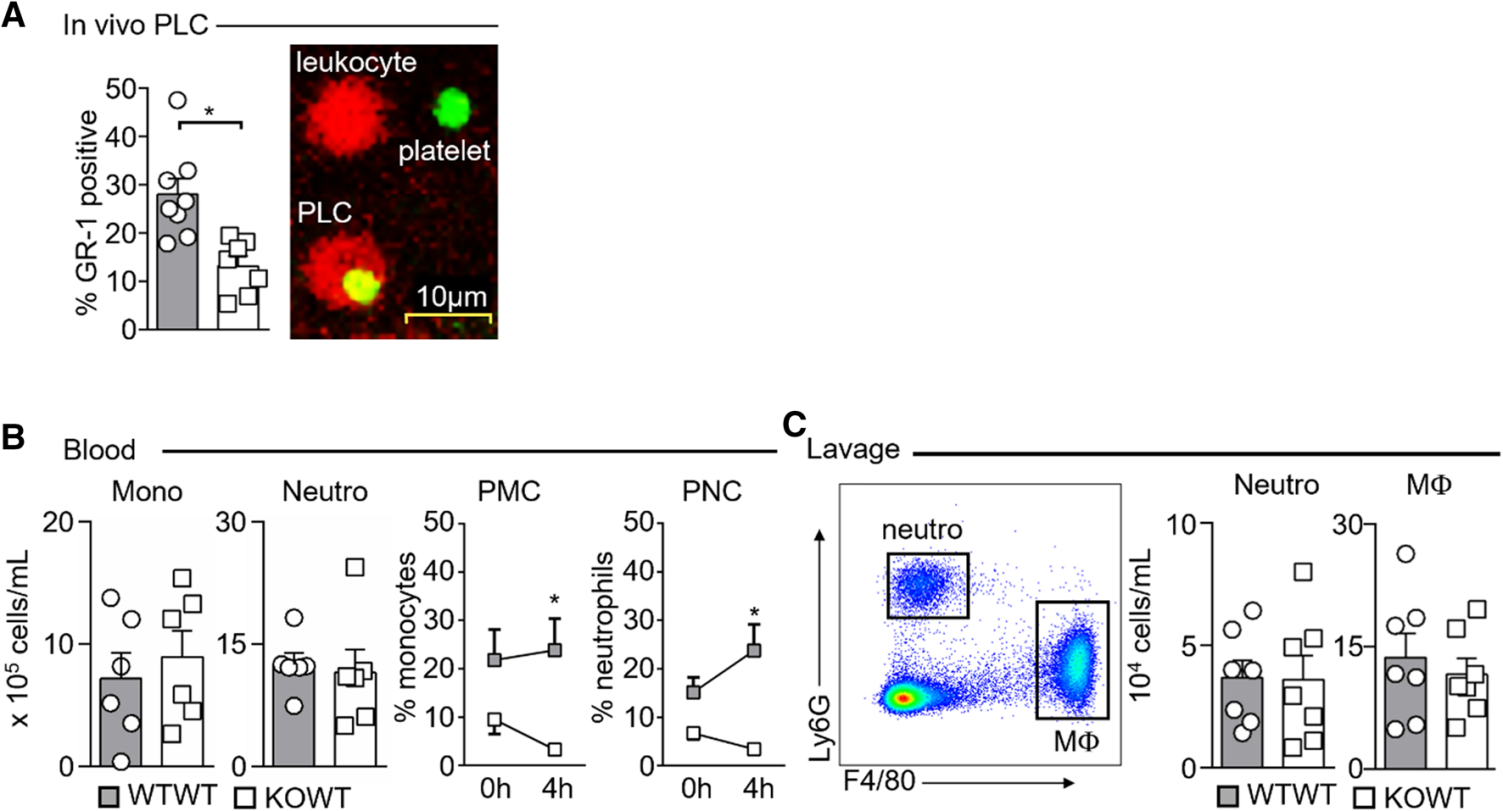
P-selectin expression on platelets is dispensable for leukocyte recruitment to sites of sterile inflammation. **A** In vivo quantification of the percentage of rolling platelet–leukocyte complexes (PLC, see representative image) in mesenteric venules of WTWT and KOWT chimeras 4 h post-TNFα i.p. stimulation. Results are presented as mean ± SEM; WTWT *n* = 8, KOWT *n* = 7. **B** Total blood monocyte and neutrophil counts and fractions of platelet–monocyte (PMC) and platelet–neutrophil complexes (PNC) in WTWT and KOWT chimeras undergoing TNFα stimulation i.p. Results are presented as mean ± SEM, *n* = 6 per group. *p < 0.05 denotes statistically significant changes between groups using 2-way ANOVA, Sidak’s multiple comparison test. **C** Representative dot plot showing gating strategy for peritoneal neutrophils and macrophages (left), and quantification of neutrophil and macrophage cell numbers in peritoneal lavage (right). Results are presented as mean ± SEM; *n* = 7 per group

Although platelets are generally thought to facilitate leukocyte attachment and infiltration into tissues, our initial intravital microscopy experiment suggested that platelet-derived P-selectin and PLC formation do not play a relevant role in this process. To substantiate our finding, we performed two-color intravital microscopy with anti-Gr-1-PE (labeling both Ly6C^high^ monocytes and neutrophils) and anti-GP1b-Alexa488 (labeling platelets) antibodies visualizing rolling PLC in vivo. Similar to the differences observed in peripheral blood, the fraction of monocytes and neutrophils rolling on or adhering to mesenteric venules which carried platelets was significantly reduced in KOWT chimeras compared to WTWT chimeras despite equal leukocyte and platelet counts (Fig. 3A, Suppl Fig. 2).

Next, we induced thioglycolate-elicited sterile peritonitis to test leukocyte infiltration in the presence or absence of PLC. 4 h post-intraperitoneal thioglycolate injection, total monocyte and neutrophil numbers were similar in blood of WTWT and KOWT chimeras, whereas PMC and PNC fractions remained significantly reduced in KOWT chimeras (Fig. 3B). Yet, the numbers of neutrophils and macrophages retrieved from the peritoneal lavage did not differ between the groups (Fig. 3C). Taken together, our experiments indicate that P-selectin-mediated leukocyte binding and PLC formation do not support inflammation-driven leukocyte extravasation.

### Elevated PLC are associated with, but dispensable for myocardial I/R injury

**Fig. 4:**
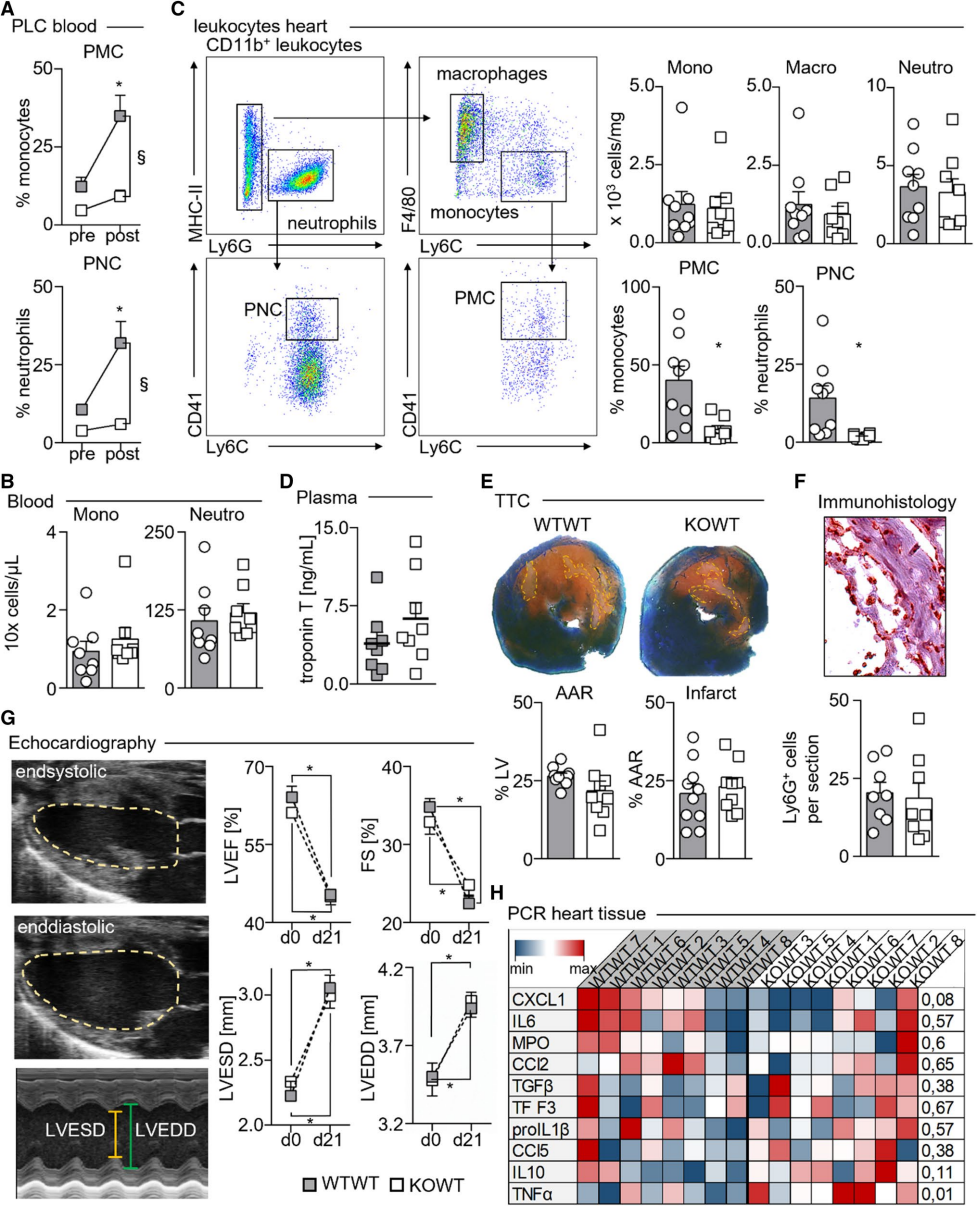
PLC have no major impact on infarct size and cardiac function post-myocardial infarction. **A** Fractions of PMC, and PNC before and 24 h after myocardial I/R injury in WTWT and KOWT chimeras. WTWT chimeras, *p < 0.05 denote statistically significant differences between time points within the same group and ^§^*p* < 0.05 between groups at the same time point. Results are presented as mean ± SEM, *n* = 8 per group, two-way ANOVA). **B** Blood counts for monocytes (Mono) and neutrophils (Neutro) 24 h after myocardial I/R injury. Results are presented as mean ± SEM, *n* = 8 per group. **C** Representative dot plots showing gating for leukocyte populations in enzymatically digested infarcted myocardium (left) and quantification of Ly6C^high^ monocytes, neutrophils, PMC and PNC, and macrophages. Results are presented as mean ± SEM, *p < 0.05 denote statistically significant differences between groups, *n* = 9 for WTWT and n = 8 for KOWT, *t* test). **D** High-sensitive Troponin T plasma levels in WTWT and KOWT chimeras 24 h after myocardial I/R injury. Results are presented as mean ± SEM, *n* = 8 per group, *t* test. **E** Representative images of left ventricle (LV) sections stained with 2,3,5-triphenyltetrazolium chloride (TTC) and quantification of the area at risk (AAR) and the infarcted area within (infarct). Results are presented as mean ± SEM, *n* = 10 for WTWT, *n* = 9 for KOWT. **F** Representative image of anti-Ly6G stained infarct areas and quantification of mean Ly6G + neutrophil numbers per LV section (ten sections analyzed per mouse). Results are presented as mean ± SEM, *n* = 8 per group, *t* test. **G** Echocardiographic analysis (representative pictures of B- and M-Mode for left-ventricular end-systolic and end-diastolic dimensions depicted) before and 21 days after myocardial I/R injury in WTWT and KOWT chimeras. Results are presented as mean ± SEM, *n* = 5 per group at d0, and *n* = 8 per group at d21, Two-way ANOVA). **H** Heat map depicting qRT-PCR-based expression levels of key genes relative to mean values of the respective genes in WTWT chimeras (blue = lowest, white = mean; red = highest gene expression) *n* = 8 per group; *p* values calculated with multiple *t*-testing and Bonferroni correction, and *p* < 0.05 denoting statically significant differences between groups

As shown in Fig. 1B, experimental myocardial I/R injury leads to a transient increase in circulating PLC numbers peaking after 24 h of reperfusion. In KOWT chimeras, however, the surge in PMC and PNC numbers observed in WTWT chimeras post-infarction does not occur (Fig. 4A) despite comparable monocyte and neutrophil numbers (Fig. 4B). In accord with our previous observations in the intravital microscopy and peritonitis models, monocyte, neutrophil, and macrophage numbers in myocardial infarct tissues, as determined by enzymatic tissue digestion and flow cytometric analysis, were similar in KOWT and WTWT chimeras, although PMC and PNC fractions were relatively reduced both in blood and myocardial tissue cell suspensions of KOWT chimeras (Fig. 4C). Immunhistologic staining for Ly6G^+^ neutrophils confirmed this finding.

Troponin T levels in plasma of KOWT and WTWT chimeras collected 24 h post-myocardial infarction showed no difference between the groups (Fig. 4D). In line, both the area at risk (AAR) and the infarct size determined by TTC staining were indistinguishable in both groups (Fig. 4E). Echocardiography revealed comparable declines in cardiac function at 21 days post-myocardial infarction in both groups (Fig. 4G). Expression levels of proinflammatory and proresolving genes in infarct tissues after 24 h of reperfusion documented only marginal differences between WTWT and KOWT chimeras (Fig. 4G). Taken together, although PLC increase as a result of myocardial I/R injury, their loss, reciprocally, does not impede myocardial injury in the short and long term.

## Discussion

Associations of elevated circulating PLCs with myocardial infarct size and adverse long-term outcomes in patients have led to the hypothesis that these two phenomena are causally related in the sense that PLC aggravate cardiac injury [47, 60, 61]. Experimental studies in the past have lent support to this hypothesis.

For instance, simultaneous infusion of neutrophils and platelets during reperfusion of ischemic isolated guinea pig and rat hearts impaired cardiac function [20, 29, 49]. In line, blocking PNC-formation attenuated these adverse cardiac effects [21]. Specifically, blocking P-selectin–P-selectin glycoprotein ligand 1 (PSGL 1) interaction reduced PLC formation, neutrophil infiltration and experimental myocardial ischemia reperfusion injury [5, 30, 41]. However, in contrast to our bone marrow chimera model, these approaches did not selectively target platelet-derived P-selectin, and thus affected endothelial P-selectin-mediated leukocyte rolling, directly.

At the site of experimental vascular injury, platelets cover the area of endothelial denudation, and platelet-derived P-selectin can imitate endothelial-derived P-selectin’s role to capture PSGL-1 expressing leukocytes and facilitate monocyte/macrophage accumulation in the neointima [33]. Similar processes may be at play during atherogenesis, when P-selectin deficiency in platelets partially protects from atherosclerotic plaque development in ApoE-deficient mice, whereas endothelial P-selectin deficiency has a more profound atheroprotective effect [8]. In myocardial infarction, however, myocardial injury develops in a large ischemic area distal to the site of an occluding coronary artery plaque, and leukocyte infiltration into the infarct tissue occurs via post-capillary venules distinct from arterial recruitment in atherosclerosis [15, 35]. Hence, mesenteric venule intravital microscopy and sterile peritonitis are valid complementary models for gaining insight into inflammatory cell recruitment into infarct tissue. In our work, intravital microscopy, peritoneal lavage analysis, and flow cytometric or immunohistochemical staining of myocardial infarct tissue showed unanimously that loss of platelet P-selectin neither slowed cell recruitment nor protected from myocardial ischemia and reperfusion injury despite a significant reduction in circulating, rolling, and tissue infiltrating PLC. In line, Rosenkranz and colleagues, using P-selectin-deficient bone marrow chimeras, reported similar kidney infiltrating leukocyte counts in a murine model of glomerulonephritis [45].

Our findings suggest a reverse causation between PLC and myocardial ischemia and reperfusion injury. Larger infarcts, as indicated by the surrogate parameter cardiac troponin, lead to the release of tissue injury related danger signals that activate platelets. Upon activation, platelets expose P-selectin forming complexes with neutrophils and monocytes. To this effect, PLC represents a biomarker of cardiac injury rather than a culprit.

Observational studies documented that elevated PLC correlate with inadequate reperfusion post-STEMI-percutaneous coronary intervention due to microvascular obstructions as assessed by reduced Thrombolysis in Myocardial Infarction (TIMI) flow and myocardial blush grade [4, 44]. A causal relationship, however, has not been established experimentally. While our study clearly indicates that PLC do not contribute to myocardial ischemia and reperfusion injury, the murine transient LAD ligation model may not fully recapitulate all pathophysiological aspects of interventional revascularization in STEMI patients, including those that lead to microvascular obstructions. Moreover, our study does not dispute a critical role of platelets in fostering leukocyte activation and extravasation by other means than P-selectin-dependent PLC formation, or in different disease contexts [48].

The biological role of PLC formation upon danger signal recognition may be immunologic. Platelets can scavenge, immobilize, and aggregate bacteria, and thus facilitate their clearance and destruction through phagocytes and neutrophil extracellular trap formation [50].

Platelets appear to circulate in the bloodstream as “surveillance drones” to be the first cellular actors on site in the context of vascular injury or tissue inflammation [31]. Healthy endothelium prevents platelet activation and aggregation, in part via nitric oxide and prostacyclin secretion. In the context of endothelial injury and activation, these inhibitory effects are compromised and platelets bind to subendothelial extracellular matrix via direct platelet GP VI–collagen interaction or von-Willebrand factor (vWF)-mediated binding to platelet GP Ib/IX/V surface complex [26, 28]. In addition, multiple soluble damage-associated molecular patterns (DAMPs) similar to pathogen-associated molecular patterns (PAMPs) activate platelets via pattern recognition receptors (Toll-like receptors, NOD-like receptors, and receptor for advanced glycation end products) [2, 13, 17, 51, 54]. Stimulated platelets activate surface integrins and release chemokines and serotonin from granules [54]. Platelet serotonin, in turn, stimulates neutrophil rolling and adhesion to the endothelium and neutrophil activation, including increased PNC formation [12, 36]. Platelet-secreted chemokines include CXCL1, platelet factor 4 (PF4/CXCL4), macrophage inflammatory protein (MIP) 1 alpha (alias CCL3), and RANTES (alias CCL5), and attract leukocytes to sites of inflammation and injury, where platelets were activated [6, 10, 19, 54]. Beyond P-selectin–PSGL-1 interaction, platelet GPIb can bind to leukocyte integrin Mac-1, directly. GPIb–Mac-1 interaction induced tissue factor expression in monocytes [55], which was not affected by platelet P-selectin deficiency and impaired PLC formation in KOWT compared to WTWT chimeras in our study (data not shown).

Both platelet-released factors and PLC formation were reported to activate leukocyte integrins [23, 56, 57]. Integrin surface expression levels on monocytes and neutrophils in mice lacking P-selectin on platelets were not altered in our experiments, although the flow cytometry antibodies may not adequately distinguish between different integrin activation statuses. Functionally, however, integrin-dependent leukocyte rolling and adhesion were not impaired in KOWT mice lacking platelet-derived P-selectin. Having said this, soluble P-selectin, which is mainly shed from activated endothelium, can still activate leukocytes via PSGL-1 [37, 52, 58] without requiring platelet-derived P-selectin and PLC formation. In line, soluble P-selectin levels were comparable in KOWT and WTWT chimeras in our study, confirming that the endothelium is the major source of soluble P-selectin even post-myocardial infarction.

Taken together, our work shows that PLC form in response to myocardial ischemia and reperfusion injury without influencing myocardial injury in return. PLC formation itself appears dispensable for key platelet and leukocyte functions in the context of myocardial injury.

## Conclusion

Platelet–leukocyte complexes form in the setting of inflammation, including sterile inflammation in the context of myocardial infarction. Preventing P-selectin-dependent complex formation, however, does not improve myocardial salvage or mitigate inflammation and leukocyte transmigration. Although PLC levels may serve as a biomarker of the magnitude of myocardial ischemia and reperfusion injury, our study suggests that targeting PLC formation, specifically, does not hold a therapeutic promise in the context of myocardial infarction.

## Supplementary Information

Below is the link to the electronic supplementary material.

Supplementary file1 (PDF 702 KB)

## Data Availability

Data are available from the corresponding author upon reasonable request.

## Abbreviations

AAR: Area at risk
ADP: Adenosindiphosphate
AMI: Acute myocardial infarction
ANOVA: One-way analysis of variance
AP: Angina pectoris
ATP: Adenosintriphosphate
Bl6J: Short for C57Bl/Bl6J
CT: Cycle treshold
CCL: C-C motif ligand
CXCL: C-X-C motif ligand
FACS: Fluorescent activated cell sorter
FS: Fractional shortening
GP: Glycoprotein
hsTnT: High-sensitive Troponin T
i.p.: Intraperitoneal
i.v.: Intravenous
I/R: Ischemia and reperfusion
IRS: Ischemia/reperfusion injury
IVM: Intravital microscopy
KO: Knock out
KOWT: Knock out in wild-type chimera
LAD: Left anterior descending
LVEDD: Left-ventricular end-diastolic diameter
LVEF: Left-ventricular ejection fraction
LVESD: Left ventricular end-systolic diameter
MFI: Mean fluorescence intensity
MIP: Macrophage inflammatory protein
Mono: Monocyte
MФ: Macrophage
neutro: Neutrophile
NSTEMI: Non-ST-elevating myocardial infarction
PBS: Phosphate buffered saline
PF4: Platelet factor 4
PLC: Platelet leukocyte complexes
PLT: Platelet/thrombocyte
PMC: Platelet monocyte complexes
PNC: Platelet neutrophile complexes
PRP: Platelet-rich plasma
RANTES: Regulated and Normal T-cell Expressed and Secreted (= CCL5)
RBC: Red blood cell
ROS: Reactive oxygen species
STEMI: ST-elevating myocardial infarction
TIMI: Thrombolysis in Myocardial Infarction
TNFα: Tumor necrosis factor alpha
TTC: 2,3,5-Triphenyltetrazoliumchloride
WBC: White blood cell
WT: Wild type
WTKO: Wild type in knockout chimera
WTWT: Wild type in wild-type chimera
