# Supplementary material for: Elevated platelet–leukocyte complexes are associated with, but dispensable for myocardial ischemia–reperfusion injury
Source: Basic Res Cardiol. 2022 Nov 16;117(1):61. doi: 10.1007/s00395-022-00970-3 (PMC9668925; doi:10.1007/s00395-022-00970-3)
Supplement: Supplementary file 1 — Supplementary file1 (PDF 702 KB) [file 395_2022_970_MOESM1_ESM.pdf]

Supplement

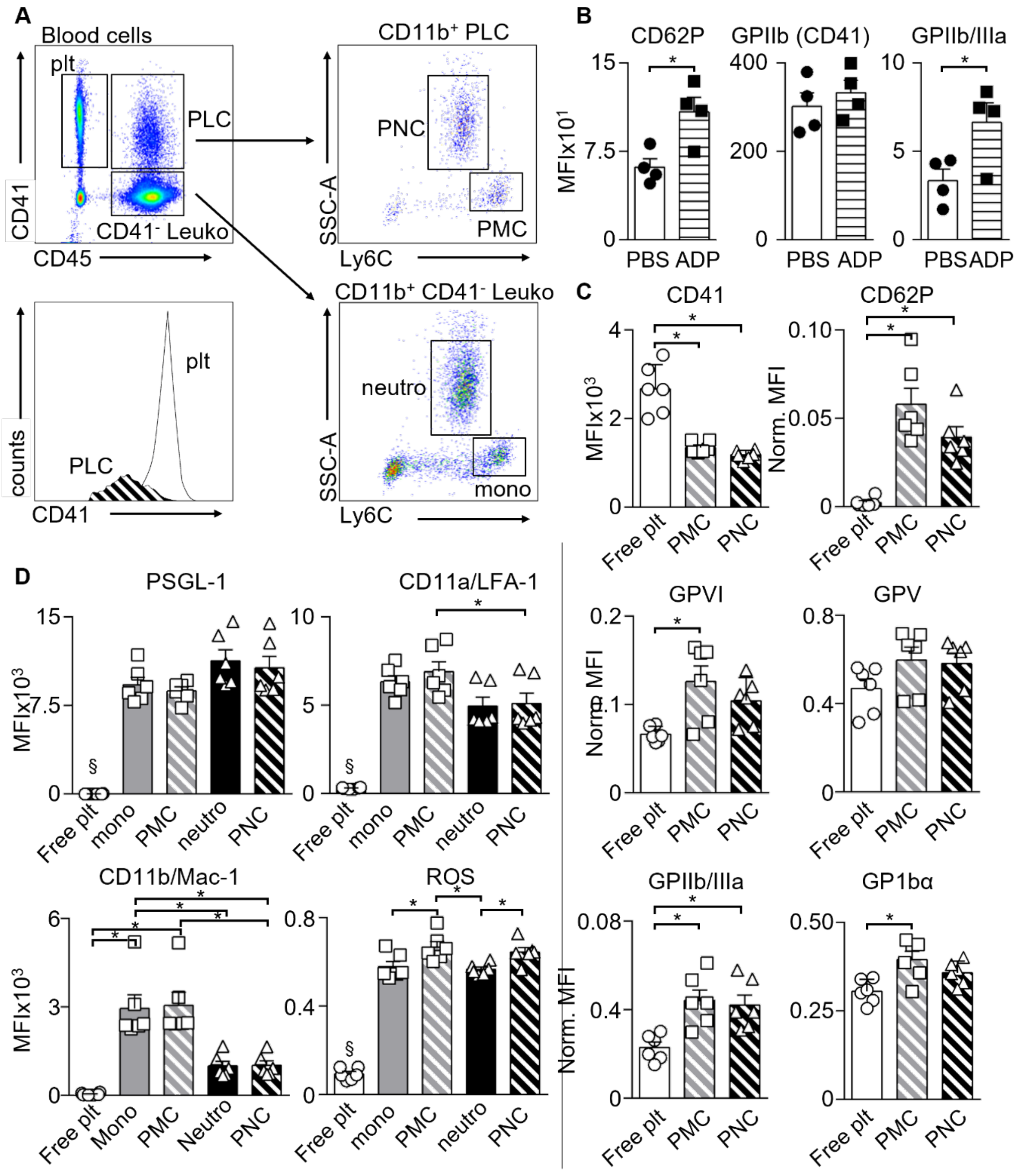

Supplemental Figure 1: A, Gating strategy to identify free and platelet-bound leukocytes (A, upper panel). Representative histogram depicting lower mean CD41 expression in platelets attached to leukocytes compared to those not attached (A, lower panel). **B,** Platelets stimulated with ADP (200nM) versus PBS for 30 min show higher P-selectin (CD62P) and activated GPIIb/IIIa receptor surface expressions whereas GPIIb (CD41) remains stable. Results are shown as mean±SEM; n=4 per group. *p<0.05, t-test. **C**, Mean fluorescence intensity (MFI) of CD41, and MFIs of CD62P, GPIIb/IIIa, GPV, GPVI, GPIb surface expressions normalized to the respective CD41 MFI on platelets with or without complex formation with neutrophils (PNC) or monocytes (PMC). Results are shown as mean±SEM; n=6 per group. *p<0.05 denotes statistically significant differences, one-way ANOVA, Holm-Sidak multiple

comparison test. **D**, MFI of PSGL-1, CD11a, CD11b surface expression levels on platelet-bound monocytes and neutrophils, and on unbound platelets, monocytes and neutrophils. MFI of DCFDA stained intracellular reactive oxygen species (ROS) activity in non-leukocyte bound platelets, monocytes, neutrophils, PMC and PNC. Results are shown as mean±SEM; n=6 per group. *p<0.05 denotes statistically significant differences, §p<0.05 denotes statistically significant differences with all shown groups, one-way ANOVA, Holm-Sidak's multiple comparison test.

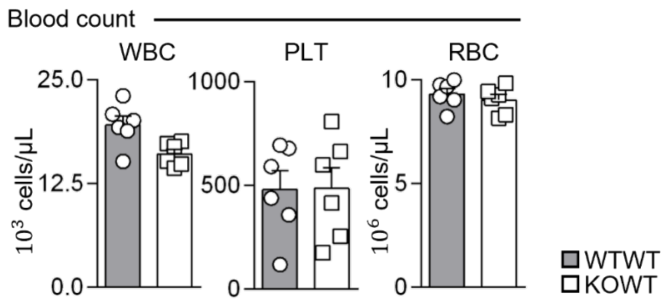

Supplemental Figure 2: Results of blood counts from WTWT and KOWT chimeras. There were no differences in cell counts. Results are presented as mean ± SEM; n=6 per group. PLT, platelets. WBC, white blood cells/leukocytes. RBC, red blood cells/erythrocytes.

Supplemental Table 1

Epitope	Conjugate	Clone	Manufacturer	Location
CD115(c-fms)	APC	AFS98	eBioscience	San Diego, CA, USA
CD11a/CD18 (LFA-1)	PerCP/Cy5.5	H155-78	BioLegend	San Diego, CA, USA
CD11b	APC/Cy7	M1/70	BD Pharmingen	San Jose, CA, USA
CD16/CD32	-	93	eBioscience	San Diego, CA, USA
CD162	BV421	2PH1	BD Bioscience	San Jose, CA, USA
CD19	PE	eBio1D3	eBioscience	San Diego, CA, USA
CD31	Pacific Blue	390	BioLegend	San Diego, CA, USA
CD41	PE/Cy7	MWreg30	BioLegend	San Diego, CA, USA
CD42d	PerCP/Cy5.5	1C2	BioLegend	San Diego, CA, USA
CD45.2	eFluor450	104	eBioscience	San Diego, CA, USA
CD45.2	Brilliant Violet 510	104	BioLegend	San Diego, CA, USA
CD45R/B220	PE	RA36B2	BD Bioscience	San Jose, CA, USA
CD49b	APC	HMα2	BioLegend	San Diego, CA, USA
CD62P	PerCP-eFlour710	Psel.K02.3	eBioscience	San Diego, CA, USA
CD62P	BV421	RB40.34	BD Horizon	San Jose, CA, USA
F4/80	PE/Cy7	BM8	BioLegend	San Diego, CA, USA
GPIbα (CD42b)	DyLight649	Xia.G5	emfret Analytics	Würzburg, Germany
GPIIb/IIIa	PE	JON/A	emfret Analytics	Würzburg, Germany
GPVI	FITC	JAQ1	emfret Analytics	Würzburg, Germany
Gr-1	PE	RB6-8C5	BioLegend	San Diego, CA, USA
Ly6C	PerCP/Cy5.5	HK1.4	BioLegend	San Diego, CA, USA
Ly6C	FITC	HK1.4	BioLegend	San Diego, CA, USA
Ly6G	PE	1A8	BD Pharmingen	San Jose, CA, USA
Ly6G	PE	1A8	BD Bioscience	San Jose, CA, USA

Supplemental Table 2

Gene	Assay ID
TNFa	Mm00443258
CCL5	Mm01302427m1
IL-6	Mm00446190m1
CCL2	Mm00441242m1
IL-1b	Mm00434226m1
TGFß	Mm01178820m1
IL-10	Mm00439614m1
CXCL1	Mm04207460m1
MPO	Mm01298424m1
F3	Mm00438855m1
β-actin	Mm4352341Em1
